# Crystal structure of tris­(*N*-methyl­salicylaldiminato-κ^2^ *N*,*O*)vanadium(III)

**DOI:** 10.1107/S2056989015021453

**Published:** 2015-11-18

**Authors:** Jessica Hilbert, Sven Kabus, Christian Näther, Wolfgang Bensch

**Affiliations:** a Institut für Anorganische Chemie, Christian-Albrechts-Universität zu Kiel, Max-Eyth-Strasse 2, 24118 Kiel, Germany

**Keywords:** crystal structure, vanadium(III), *N*-methyl­salicylaldiminate

## Abstract

The structure of the title complex, [V(C_8_H_8_NO)_3_], comprises neutral and discrete complexes, in which the V^III^ cation is coordinated by three anionic *N*-methyl­alicylaldiminate ligands within a slightly distorted *mer*-N_3_O_3_ octa­hedral geometry. In the crystal structure, the mol­ecules are linked *via* C—H⋯O hydrogen bonds into supra­molecular chains that extend along the *c* axis.

## Related literature   

For structures of discrete complexes of Mo and V with *N*-methyl­saldicylaldiminate as the ligand, see: Davies & Gatehouse (1974 ▸); Cornman *et al.* (1997 ▸). For the synthesis of the starting material, see: Bonadies & Carrano (1986 ▸).
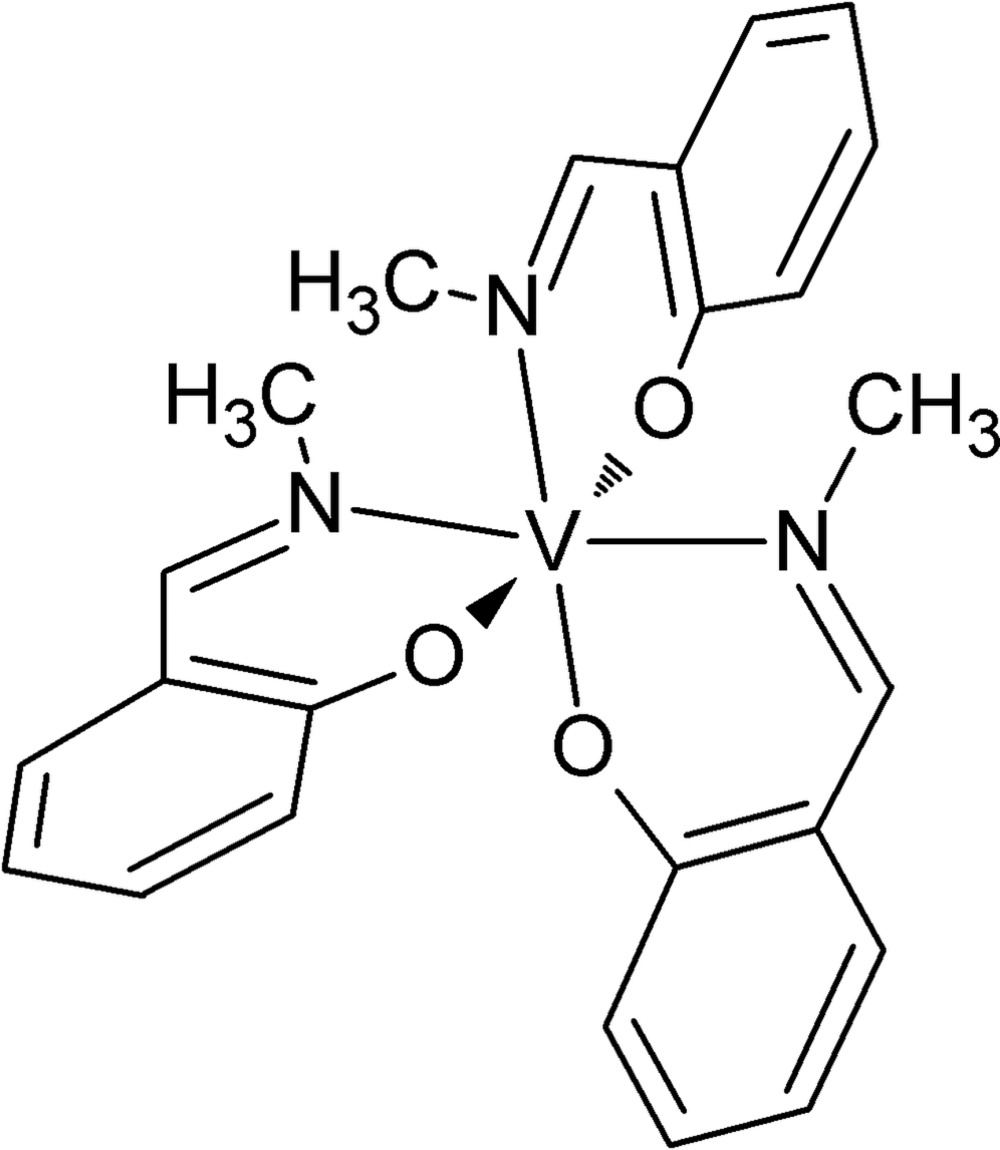


## Experimental   

### Crystal data  

[V(C_8_H_8_NO)_3_]
*M* *_r_* = 453.40
Monoclinic, 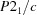

*a* = 7.7414 (3) Å
*b* = 26.0018 (7) Å
*c* = 11.1004 (4) Å
β = 103.265 (3)°
*V* = 2174.79 (13) Å^3^
*Z* = 4
Mo *K*α radiation
μ = 0.49 mm^−1^
*T* = 170 K
0.24 × 0.14 × 0.06 mm

### Data collection  

STOE IPDS-1 diffractometer
Absorption correction: numerical (*X-SHAPE* and *X-RED32*; Stoe, 2008 ▸) *T*_min_ = 0.919, *T*_max_ = 0.974
18648 measured reflections
4741 independent reflections
4054 reflections with *I* > 2σ(*I*)
*R*_int_ = 0.030

### Refinement  

*R*[*F* ^2^ > 2σ(*F* ^2^)] = 0.040
*wR*(*F* ^2^) = 0.102
*S* = 1.07
4741 reflections
283 parameters
H-atom parameters constrained
Δρ_max_ = 0.30 e Å^−3^
Δρ_min_ = −0.42 e Å^−3^

### 

Data collection: *X-AREA* (Stoe, 2008 ▸); cell refinement: *X-AREA*; data reduction: *X-AREA*; program(s) used to solve structure: *SHELXS97* (Sheldrick, 2008 ▸); program(s) used to refine structure: *SHELXL2014* (Sheldrick, 2015 ▸); molecular graphics: *XP* in *SHELXTL* (Sheldrick, 2008 ▸) and *DIAMOND* (Brandenburg, 1999 ▸); software used to prepare material for publication: *publCIF* (Westrip, 2010 ▸).

## Supplementary Material

Crystal structure: contains datablock(s) I, global. DOI: 10.1107/S2056989015021453/tk5408sup1.cif

Structure factors: contains datablock(s) I. DOI: 10.1107/S2056989015021453/tk5408Isup2.hkl

**Fig. 1.**
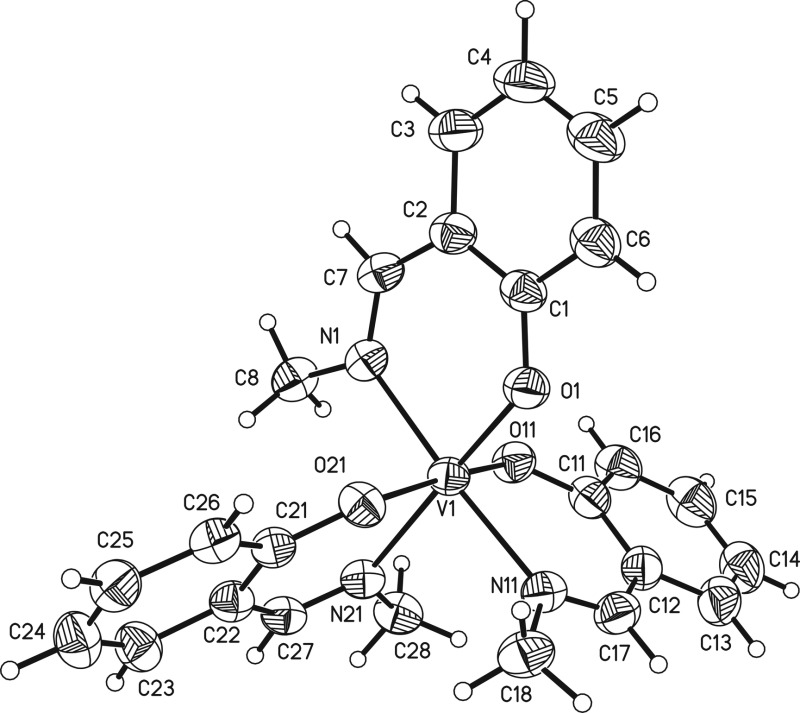
The molecular structure of the title complex with atom labelling. Displacement ellipsoids are drawn at the 50% probability level.

**Fig. 2.**
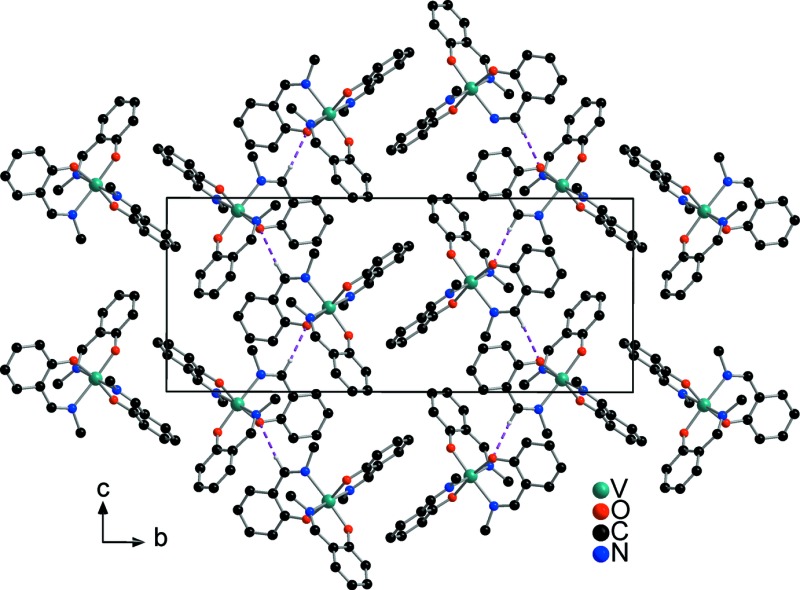
Unit cell contents of the crystal structure of the title complex viewed in projection down the a axis with hydrogen bonds shown as dashed lines. For clarity, all H atoms except those that participates in hydrogen bonding are omitted.

. DOI: 10.1107/S2056989015021453/tk5408fig1.tif
The mol­ecular structure of the title complex with atom labelling. Displacement ellipsoids are drawn at the 50% probability level.

a . DOI: 10.1107/S2056989015021453/tk5408fig2.tif
Unit cell contents of the crystal structure of the title complex viewed in projection down the *a* axis with hydrogen bonds shown as dashed lines. For clarity, all H atoms except those that participates in hydrogen bonding are omitted.

CCDC reference: 1436532

Additional supporting information:  crystallographic information; 3D view; checkCIF report

## Figures and Tables

**Table 1:** Hydrogen-bond geometry (Å, °)

*D*—H⋯*A*	*D*—H	H⋯*A*	*D*⋯*A*	*D*—H⋯*A*
C27—H27⋯O21^i^	0.95	2.56	3.431 (2)	153

Symmetry code: (i) 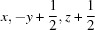.

## supplementary crystallographic information

### S1. Synthesis and crystallization

Most of the chemicals are commercially available: Sn (Fluka, 99.9%), S (Alfa Aesar, 99.5%), and methyl­amine (abcr, 40% aqueous solution). (*N*,*N*'-Disalicyl­idene­ethyl­enedi­amine)­oxovanadium(IV) was prepared following the procedure of Bonadies & Carrano (1986): (*N*,*N*'-disalicyl­idene­ethyl­enedi­amine)­oxovanadium (IV) (83.8 mg, 0.25 mmol ), Sn (29.7 mg, 0.25 mmol) and S (24.1 mg, 0.75 mmol) were reacted in a glass tube (inner volume 11 mL) with methyl­amine (1.5 mL) and H_2_O (0.5 mL) under solvothermal conditions at 120 °C for 24 h. Afterwards, the solid residue was filtered off, washed with water and ethanol, and dried over silica gel. The product contains red blocks of the title complex and a small amount of brown blocks of bis­(*N*-methyl­saldicylaliminato)oxovanadium(IV) (Cornman *et al.* (1997)). Even if Sn and S are not contained in the final product, they are needed for product formation, as otherwise only (*N*,*N*'-disalicyl­idene­ethyl­enedi­amine)­oxovanadium(IV) is isolated.

### S2. Refinement

The C—H H atoms were positioned with idealized geometry (methyl H atoms allowed to rotate but not to tip) and were refined isotropically with *U*_eq_(H) = 1.2 *U*_eq_(C) (1.5 for methyl H atoms) using a riding model with C—H = 0.95 Å for aromatic H atoms and 0.98 Å for methyl H atoms.

### Crystal data

**Table:**

[V(C_8_H_8_NO)_3_]	*F*(000) = 944
*M**_r_* = 453.40	*D*_x_ = 1.385 Mg m^−^^3^
Monoclinic, *P*2_1_/*c*	Mo *K*α radiation, λ = 0.71073 Å
*a* = 7.7414 (3) Å	Cell parameters from 18648 reflections
*b* = 26.0018 (7) Å	θ = 1.6–27.0°
*c* = 11.1004 (4) Å	µ = 0.49 mm^−^^1^
β = 103.265 (3)°	*T* = 170 K
*V* = 2174.79 (13) Å^3^	Block, red
*Z* = 4	0.24 × 0.14 × 0.06 mm

### Data collection

**Table:**

STOE IPDS-1 diffractometer	4054 reflections with *I* > 2σ(*I*)
Radiation source: fine-focus sealed tube	*R*_int_ = 0.030
φ–scans	θ_max_ = 27.0°, θ_min_ = 1.6°
Absorption correction: numerical (*X-SHAPE* and *X-RED32*; Stoe, 2008)	*h* = −9→9
*T*_min_ = 0.919, *T*_max_ = 0.974	*k* = −30→33
18648 measured reflections	*l* = −14→14
4741 independent reflections	

### Refinement

**Table:**

Refinement on *F*^2^	0 restraints
Least-squares matrix: full	Hydrogen site location: inferred from neighbouring sites
*R*[*F*^2^ > 2σ(*F*^2^)] = 0.040	H-atom parameters constrained
*wR*(*F*^2^) = 0.102	*w* = 1/[σ^2^(*F*_o_^2^) + (0.0523*P*)^2^ + 0.6659*P*] where *P* = (*F*_o_^2^ + 2*F*_c_^2^)/3
*S* = 1.07	(Δ/σ)_max_ = 0.001
4741 reflections	Δρ_max_ = 0.30 e Å^−^^3^
283 parameters	Δρ_min_ = −0.42 e Å^−^^3^

### Special details

**Table:**

Geometry. All esds (except the esd in the dihedral angle between two l.s. planes) are estimated using the full covariance matrix. The cell esds are taken into account individually in the estimation of esds in distances, angles and torsion angles; correlations between esds in cell parameters are only used when they are defined by crystal symmetry. An approximate (isotropic) treatment of cell esds is used for estimating esds involving l.s. planes.

### Fractional atomic coordinates and isotropic or equivalent isotropic displacement parameters (Å^2^)

**Table:**

	*x*	*y*	*z*	*U*_iso_*/*U*_eq_	
V1	0.60947 (4)	0.34777 (2)	0.43424 (3)	0.03210 (10)	
O1	0.58715 (17)	0.39024 (5)	0.28969 (12)	0.0400 (3)	
C1	0.6728 (2)	0.39169 (7)	0.19994 (17)	0.0375 (4)	
C2	0.8151 (3)	0.35824 (8)	0.19485 (18)	0.0390 (4)	
C3	0.8995 (3)	0.36297 (9)	0.0951 (2)	0.0497 (5)	
H3	0.9964	0.3410	0.0919	0.060*	
C4	0.8448 (3)	0.39846 (10)	0.0029 (2)	0.0578 (6)	
H4	0.9030	0.4010	−0.0634	0.069*	
C5	0.7033 (3)	0.43068 (10)	0.0075 (2)	0.0550 (6)	
H5	0.6646	0.4552	−0.0564	0.066*	
C6	0.6186 (3)	0.42752 (9)	0.10360 (19)	0.0466 (5)	
H6	0.5221	0.4499	0.1049	0.056*	
C7	0.8789 (2)	0.31882 (8)	0.28513 (17)	0.0384 (4)	
H7	0.9766	0.2989	0.2733	0.046*	
N1	0.81716 (19)	0.30777 (6)	0.38049 (14)	0.0351 (3)	
C8	0.9074 (3)	0.26689 (8)	0.46168 (18)	0.0423 (4)	
H8A	0.9545	0.2808	0.5448	0.064*	
H8B	1.0053	0.2533	0.4287	0.064*	
H8C	0.8231	0.2392	0.4659	0.064*	
O11	0.78967 (17)	0.38679 (5)	0.54398 (12)	0.0397 (3)	
C11	0.7839 (3)	0.42940 (8)	0.60833 (18)	0.0402 (4)	
C12	0.6228 (3)	0.45333 (8)	0.61828 (18)	0.0416 (4)	
C13	0.6290 (3)	0.49804 (9)	0.6899 (2)	0.0510 (5)	
H13	0.5211	0.5143	0.6952	0.061*	
C14	0.7869 (4)	0.51892 (9)	0.7524 (2)	0.0580 (6)	
H14	0.7882	0.5490	0.8012	0.070*	
C15	0.9450 (4)	0.49555 (10)	0.7434 (2)	0.0587 (6)	
H15	1.0549	0.5099	0.7863	0.070*	
C16	0.9439 (3)	0.45159 (9)	0.6727 (2)	0.0501 (5)	
H16	1.0533	0.4362	0.6676	0.060*	
C17	0.4494 (3)	0.43340 (8)	0.56016 (18)	0.0430 (4)	
H17	0.3499	0.4519	0.5740	0.052*	
N11	0.4164 (2)	0.39324 (6)	0.49186 (15)	0.0378 (3)	
C18	0.2295 (2)	0.37888 (9)	0.4467 (2)	0.0494 (5)	
H18A	0.2020	0.3762	0.3562	0.074*	
H18B	0.1538	0.4051	0.4715	0.074*	
H18C	0.2079	0.3456	0.4822	0.074*	
O21	0.42824 (16)	0.30065 (5)	0.34205 (11)	0.0370 (3)	
C21	0.3734 (2)	0.25415 (7)	0.35945 (16)	0.0328 (4)	
C22	0.4208 (2)	0.22876 (7)	0.47477 (17)	0.0352 (4)	
C23	0.3555 (3)	0.17928 (8)	0.4881 (2)	0.0446 (5)	
H23	0.3849	0.1630	0.5668	0.054*	
C24	0.2496 (3)	0.15367 (9)	0.3894 (2)	0.0499 (5)	
H24	0.2098	0.1197	0.3990	0.060*	
C25	0.2023 (3)	0.17858 (8)	0.2756 (2)	0.0439 (5)	
H25	0.1293	0.1613	0.2070	0.053*	
C26	0.2596 (2)	0.22787 (8)	0.26080 (18)	0.0376 (4)	
H26	0.2219	0.2445	0.1830	0.045*	
C27	0.5263 (2)	0.25346 (7)	0.58385 (16)	0.0349 (4)	
H27	0.5412	0.2352	0.6597	0.042*	
N21	0.60191 (18)	0.29761 (6)	0.58834 (13)	0.0342 (3)	
C28	0.6848 (3)	0.31610 (9)	0.71350 (17)	0.0426 (4)	
H28A	0.6331	0.3493	0.7276	0.064*	
H28B	0.8128	0.3201	0.7215	0.064*	
H28C	0.6637	0.2912	0.7748	0.064*	

### Atomic displacement parameters (Å^2^)

**Table:**

	*U*^11^	*U*^22^	*U*^33^	*U*^12^	*U*^13^	*U*^23^
V1	0.03170 (16)	0.03642 (17)	0.02820 (16)	−0.00124 (12)	0.00694 (11)	−0.00003 (12)
O1	0.0416 (7)	0.0421 (7)	0.0386 (7)	0.0028 (6)	0.0140 (5)	0.0045 (6)
C1	0.0413 (9)	0.0401 (10)	0.0313 (9)	−0.0078 (8)	0.0092 (7)	−0.0013 (8)
C2	0.0401 (9)	0.0438 (11)	0.0339 (9)	−0.0078 (8)	0.0101 (7)	−0.0057 (8)
C3	0.0530 (12)	0.0571 (13)	0.0436 (11)	−0.0077 (10)	0.0207 (9)	−0.0064 (10)
C4	0.0709 (15)	0.0681 (15)	0.0409 (12)	−0.0128 (12)	0.0264 (11)	−0.0010 (11)
C5	0.0711 (14)	0.0583 (14)	0.0364 (11)	−0.0081 (11)	0.0141 (10)	0.0083 (10)
C6	0.0543 (11)	0.0454 (11)	0.0398 (10)	−0.0047 (9)	0.0105 (9)	0.0034 (9)
C7	0.0324 (8)	0.0467 (11)	0.0369 (10)	−0.0013 (8)	0.0098 (7)	−0.0075 (8)
N1	0.0322 (7)	0.0392 (8)	0.0325 (8)	−0.0001 (6)	0.0045 (6)	−0.0042 (7)
C8	0.0383 (9)	0.0494 (11)	0.0373 (10)	0.0086 (8)	0.0044 (8)	0.0007 (9)
O11	0.0380 (7)	0.0424 (7)	0.0389 (7)	−0.0036 (5)	0.0095 (5)	−0.0066 (6)
C11	0.0474 (10)	0.0388 (10)	0.0341 (9)	−0.0077 (8)	0.0084 (8)	−0.0013 (8)
C12	0.0548 (11)	0.0368 (10)	0.0343 (9)	0.0009 (8)	0.0121 (8)	0.0004 (8)
C13	0.0704 (14)	0.0423 (11)	0.0411 (11)	0.0029 (10)	0.0145 (10)	−0.0016 (9)
C14	0.0837 (17)	0.0436 (12)	0.0470 (12)	−0.0106 (11)	0.0156 (11)	−0.0081 (10)
C15	0.0691 (15)	0.0558 (14)	0.0484 (12)	−0.0224 (12)	0.0075 (11)	−0.0072 (11)
C16	0.0520 (11)	0.0516 (12)	0.0459 (11)	−0.0129 (10)	0.0095 (9)	−0.0056 (10)
C17	0.0460 (10)	0.0461 (11)	0.0393 (10)	0.0074 (8)	0.0148 (8)	0.0021 (9)
N11	0.0366 (8)	0.0424 (9)	0.0356 (8)	0.0021 (6)	0.0105 (6)	0.0010 (7)
C18	0.0337 (9)	0.0605 (13)	0.0550 (13)	0.0018 (9)	0.0120 (9)	−0.0024 (11)
O21	0.0391 (6)	0.0404 (7)	0.0296 (6)	−0.0041 (5)	0.0041 (5)	0.0024 (5)
C21	0.0287 (8)	0.0365 (9)	0.0333 (9)	0.0011 (7)	0.0076 (6)	−0.0002 (7)
C22	0.0317 (8)	0.0394 (10)	0.0345 (9)	0.0025 (7)	0.0074 (7)	0.0020 (8)
C23	0.0429 (10)	0.0417 (11)	0.0477 (11)	0.0003 (8)	0.0073 (8)	0.0075 (9)
C24	0.0461 (11)	0.0408 (11)	0.0605 (13)	−0.0048 (9)	0.0074 (10)	0.0008 (10)
C25	0.0375 (9)	0.0464 (11)	0.0463 (11)	−0.0026 (8)	0.0062 (8)	−0.0075 (9)
C26	0.0325 (8)	0.0456 (11)	0.0342 (9)	0.0005 (7)	0.0066 (7)	−0.0038 (8)
C27	0.0311 (8)	0.0431 (10)	0.0305 (8)	0.0038 (7)	0.0070 (6)	0.0070 (8)
N21	0.0318 (7)	0.0427 (9)	0.0271 (7)	0.0009 (6)	0.0045 (6)	0.0015 (6)
C28	0.0430 (10)	0.0553 (12)	0.0271 (9)	−0.0048 (9)	0.0032 (7)	0.0012 (9)

### Geometric parameters (Å, º)

**Table:**

V1—O11	1.9183 (13)	C13—H13	0.9500
V1—O1	1.9227 (14)	C14—C15	1.390 (4)
V1—O21	1.9641 (13)	C14—H14	0.9500
V1—N1	2.1126 (15)	C15—C16	1.386 (3)
V1—N11	2.1163 (16)	C15—H15	0.9500
V1—N21	2.1625 (15)	C16—H16	0.9500
O1—C1	1.318 (2)	C17—N11	1.281 (3)
C1—C6	1.408 (3)	C17—H17	0.9500
C1—C2	1.415 (3)	N11—C18	1.467 (2)
C2—C3	1.414 (3)	C18—H18A	0.9800
C2—C7	1.439 (3)	C18—H18B	0.9800
C3—C4	1.371 (3)	C18—H18C	0.9800
C3—H3	0.9500	O21—C21	1.310 (2)
C4—C5	1.389 (4)	C21—C22	1.412 (3)
C4—H4	0.9500	C21—C26	1.414 (2)
C5—C6	1.376 (3)	C22—C23	1.402 (3)
C5—H5	0.9500	C22—C27	1.447 (3)
C6—H6	0.9500	C23—C24	1.379 (3)
C7—N1	1.290 (2)	C23—H23	0.9500
C7—H7	0.9500	C24—C25	1.392 (3)
N1—C8	1.463 (2)	C24—H24	0.9500
C8—H8A	0.9800	C25—C26	1.379 (3)
C8—H8B	0.9800	C25—H25	0.9500
C8—H8C	0.9800	C26—H26	0.9500
O11—C11	1.325 (2)	C27—N21	1.284 (2)
C11—C16	1.404 (3)	C27—H27	0.9500
C11—C12	1.420 (3)	N21—C28	1.471 (2)
C12—C13	1.403 (3)	C28—H28A	0.9800
C12—C17	1.446 (3)	C28—H28B	0.9800
C13—C14	1.371 (3)	C28—H28C	0.9800
			
O11—V1—O1	98.00 (6)	C14—C13—H13	119.2
O11—V1—O21	171.59 (6)	C12—C13—H13	119.2
O1—V1—O21	90.39 (6)	C13—C14—C15	119.2 (2)
O11—V1—N1	87.14 (6)	C13—C14—H14	120.4
O1—V1—N1	88.60 (6)	C15—C14—H14	120.4
O21—V1—N1	92.56 (6)	C16—C15—C14	120.7 (2)
O11—V1—N11	88.49 (6)	C16—C15—H15	119.7
O1—V1—N11	89.82 (6)	C14—C15—H15	119.7
O21—V1—N11	92.09 (6)	C15—C16—C11	121.1 (2)
N1—V1—N11	175.10 (6)	C15—C16—H16	119.4
O11—V1—N21	87.96 (6)	C11—C16—H16	119.4
O1—V1—N21	173.21 (6)	N11—C17—C12	126.48 (19)
O21—V1—N21	83.70 (5)	N11—C17—H17	116.8
N1—V1—N21	94.99 (6)	C12—C17—H17	116.8
N11—V1—N21	87.06 (6)	C17—N11—C18	117.16 (17)
C1—O1—V1	133.07 (12)	C17—N11—V1	125.15 (13)
O1—C1—C6	118.64 (18)	C18—N11—V1	117.65 (13)
O1—C1—C2	122.96 (17)	N11—C18—H18A	109.5
C6—C1—C2	118.39 (18)	N11—C18—H18B	109.5
C3—C2—C1	118.79 (19)	H18A—C18—H18B	109.5
C3—C2—C7	117.42 (19)	N11—C18—H18C	109.5
C1—C2—C7	123.78 (17)	H18A—C18—H18C	109.5
C4—C3—C2	121.6 (2)	H18B—C18—H18C	109.5
C4—C3—H3	119.2	C21—O21—V1	135.69 (11)
C2—C3—H3	119.2	O21—C21—C22	122.67 (16)
C3—C4—C5	119.3 (2)	O21—C21—C26	119.81 (16)
C3—C4—H4	120.3	C22—C21—C26	117.52 (17)
C5—C4—H4	120.3	C23—C22—C21	119.98 (17)
C6—C5—C4	120.8 (2)	C23—C22—C27	117.86 (17)
C6—C5—H5	119.6	C21—C22—C27	122.03 (17)
C4—C5—H5	119.6	C24—C23—C22	121.5 (2)
C5—C6—C1	121.1 (2)	C24—C23—H23	119.3
C5—C6—H6	119.5	C22—C23—H23	119.3
C1—C6—H6	119.5	C23—C24—C25	118.7 (2)
N1—C7—C2	126.67 (18)	C23—C24—H24	120.6
N1—C7—H7	116.7	C25—C24—H24	120.6
C2—C7—H7	116.7	C26—C25—C24	121.06 (19)
C7—N1—C8	116.91 (16)	C26—C25—H25	119.5
C7—N1—V1	124.86 (13)	C24—C25—H25	119.5
C8—N1—V1	118.07 (12)	C25—C26—C21	121.15 (18)
N1—C8—H8A	109.5	C25—C26—H26	119.4
N1—C8—H8B	109.5	C21—C26—H26	119.4
H8A—C8—H8B	109.5	N21—C27—C22	126.46 (17)
N1—C8—H8C	109.5	N21—C27—H27	116.8
H8A—C8—H8C	109.5	C22—C27—H27	116.8
H8B—C8—H8C	109.5	C27—N21—C28	115.14 (16)
C11—O11—V1	132.48 (12)	C27—N21—V1	127.10 (12)
O11—C11—C16	118.88 (19)	C28—N21—V1	117.71 (12)
O11—C11—C12	123.17 (17)	N21—C28—H28A	109.5
C16—C11—C12	117.93 (19)	N21—C28—H28B	109.5
C13—C12—C11	119.37 (19)	H28A—C28—H28B	109.5
C13—C12—C17	117.2 (2)	N21—C28—H28C	109.5
C11—C12—C17	123.40 (18)	H28A—C28—H28C	109.5
C14—C13—C12	121.7 (2)	H28B—C28—H28C	109.5

### Hydrogen-bond geometry (Å, º)

**Table:**

*D*—H···*A*	*D*—H	H···*A*	*D*···*A*	*D*—H···*A*
C27—H27···O21^i^	0.95	2.56	3.431 (2)	153

Symmetry code: (i) *x*, −*y*+1/2, *z*+1/2.
