# Supplementary figures and images for: Crystal structure of tris­(N-methyl­salicylaldiminato-κ2 N,O)vanadium(III)
Source: Acta Crystallogr E Crystallogr Commun. 2015 Nov 18;71(Pt 12):m225. doi: 10.1107/S2056989015021453 (PMC4719845; doi:10.1107/S2056989015021453)

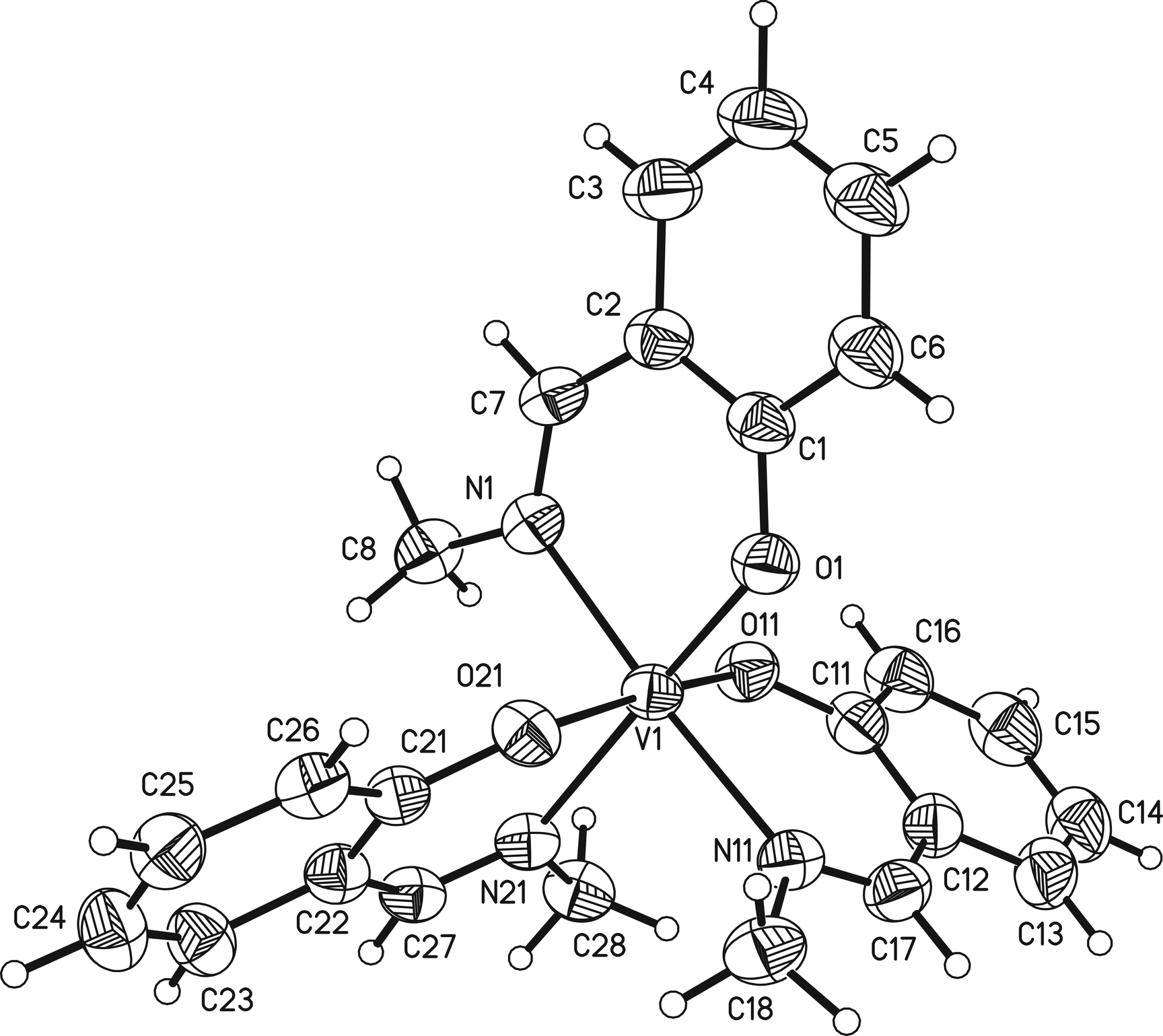

Supplement: Supplementary file 3 [file e-71-0m225-fig1.tif]

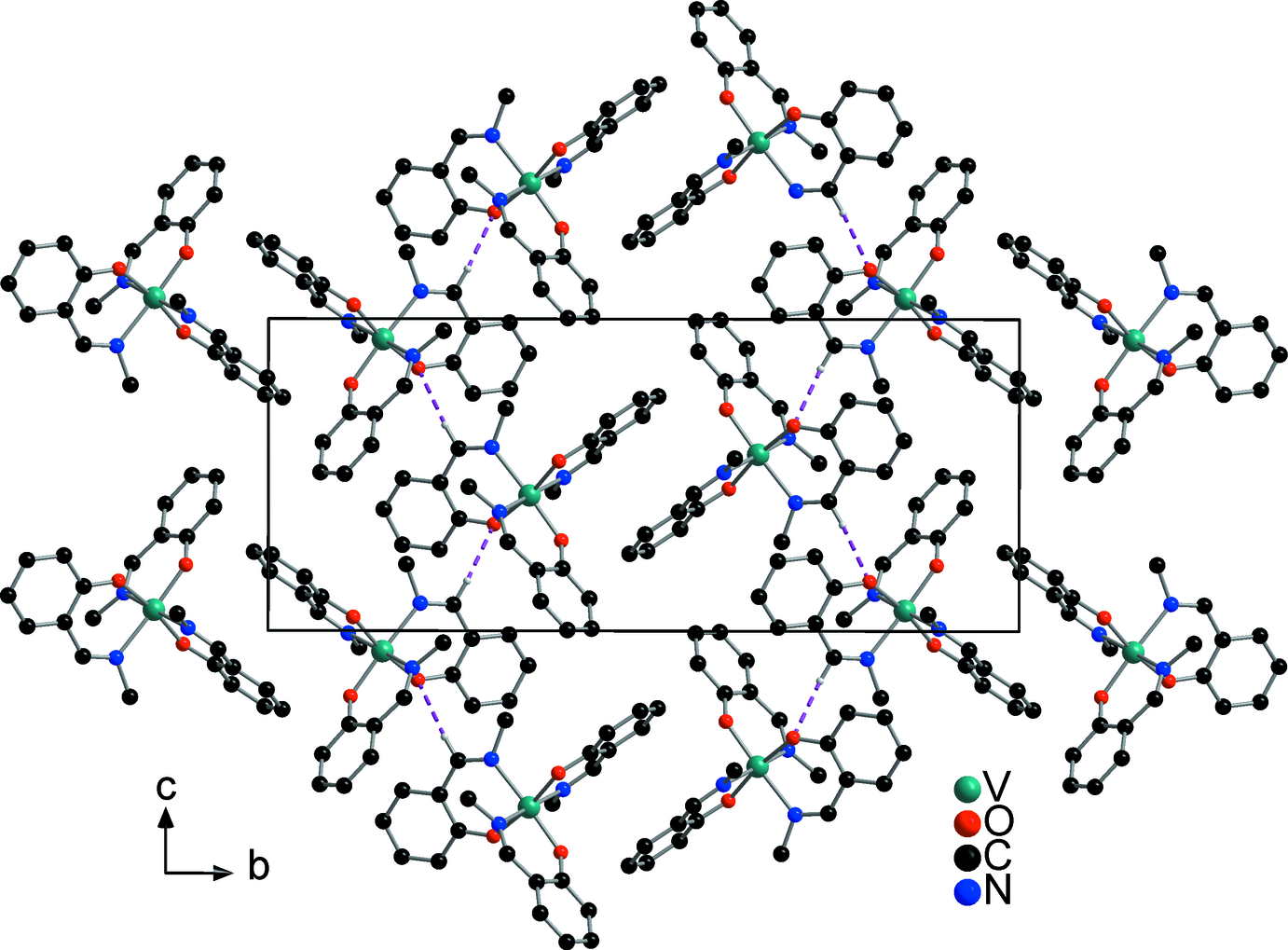

Supplement: Supplementary file 4 [file e-71-0m225-fig2.tif]
